# Treatment of distal radius fractures using a cemented K-wire frame

**DOI:** 10.1186/s12891-022-05550-z

**Published:** 2022-06-20

**Authors:** Hongyu Meng, Bin Xu, Yi Xu, Haiyun Niu, Ning Liu, Donglei Sun

**Affiliations:** 1 Department of Orthopaedic Surgery, Third Hospital of Hebei Medical University, Shijiazhuang, 050000 Hebei China; 2 Key Laboratory of Biomechanics of Hebei Province, Shijiazhuang, 050000 Hebei China; 3 Orthopaedic Research Institution of Hebei Province, Shijiazhuang, 050000 Hebei China; 4 Department of Hand Surgery, Third Hospital of Hebei Medical University, Shijiazhuang, 050051 Hebei China; 5 Central Laboratory, Second Hospital of Hebei Medical University, Shijiazhuang, 050000 Hebei China

**Keywords:** Distal radial fracture, Pining, K-wire

## Abstract

**Background:**

This retrospective study included an alternative treatment for types A2, A3, and B1 distal radius fractures using percutaneous fixation with a cemented K-wire frame.

**Methods:**

From January 2017 to January 2020, 78 patients with distal radius fractures were treated with percutaneous internal fixation using a cemented K-wire frame. There were 47 male patients and 31 female patients. The fractures were classified into types A2 (*n* = 10), A3 (*n* = 46), and B1 (*n* = 22). X-rays were taken immediately after surgery and after the bone had healed. Wrist function was assessed using the Mayo Wrist Score (90–100, excellent; 80–90, good; 60–80, satisfactory; < 60, poor). Patient satisfaction was assessed using the 10-cm visual analog scale.

**Results:**

Neither fixation failure nor K-wire migration was found (*P* > 0.05). Osteomyelitis was not observed in this series. All patients achieved bone healing after a mean of 4.5 weeks (range, 4 to 8 weeks). Follow-up lasted a mean of 27 months (range, 24 to 33 months). The mean score of wrist function was 97 (range, 91 to 100). Among them, 66 results were excellent and 12 results were good. The mean patient satisfaction was 10 cm (range, 8 to 10 cm).

**Conclusions:**

Percutaneous fixation with cemented K-wire frame is a safe and preferred choice for the treatment of types A2, A3, and B1 distal radius fractures. The frame provides support to prevent wire migration. The fixation technique is a minimally invasive procedure that is easy to perform.

**Level of Evidence:**

Therapeutic study, Level IVa.

**Supplementary Information:**

The online version contains supplementary material available at 10.1186/s12891-022-05550-z.

## Introduction

Distal radius fractures (DRFs) account for 8% − 15% of all fractures [1]. Stable DRFs are usually treated conservatively, and unstable DRFs are usually treated surgically [2]. Fixation implants include plate and screw systems, K-wires, external fixators, etc. The treatment option is based on the fracture types, soft tissue envelope injuries, articular surface involvement, surgeon preference, patient’s health status, etc [3]. Currently, the optimal technique is still debated. K-wire fixation is a widely accepted treatment, but the optimal configuration is controversial [4].

Clinical practice guidelines from the American Academy of Orthopaedic Surgeons (AAOS) moderately recommend stable surgical fixation, rather than cast immobilization, followed by early wrist movement to treat displaced DRFs [5]. Percutaneous pinning can help reduce and stabilize the fragments in a minimally invasive manner, especially in DRFs near the wrist (AO types A2 and A3) and some AO type C fractures. Ideally, the Kapandji wiring technique provides high reactive torque to reduce and maintain reduction by passing K-wires through the anatomical windows [6]. However, optimal wire placement may be difficult to achieve due to tendons, fracture patterns, damaged cortical wall, and anatomical variations [7]. A limited number of windows are invisible on the skin. Those weaknesses may lead to unstable fixation and redisplacement [8]. Adding more K-wires increases the risks of tendon irritation, tendon rupture, neurovascular injuries, and pin tract infections [9]. In addition, oblique Kapandji K-wires engaging a single cortical wall risk wire migration, especially in oblique and comminuted fractures. The stability depends on proper wire placement, fracture patterns, intact cortical walls, and good bone quality, but these factors may be affected by the inability to visualize the soft tissues and fracture site [10]. Those drawbacks prompted us to modify the fixation technique. The key is to achieve safe wire placement and prevent wire migration and fracture redisplacement.

This retrospective study included an alternative treatment for types A2, A3, and B1 DRFs with percutaneous fixation using a cemented K-wire frame. We also report the effectiveness of the novel fixation technique.

## Materials and methods

The study was approved by the Institutional Review Board of the hospital involved. Informed consent was obtained from all participants and their legal guardian(s) for the participants under the age of 18. All methods were performed in accordance with the relevant guidelines and regulations.

From January 2017 to January 2020, 78 patients with DRFs were treated with percutaneous fixation using a cemented K-wire frame. The eligibility criteria for the study were as follows: age ≥ 16 or < 70 years old; closed DRFs; AO/OTA (AO Foundation and Orthopaedic Trauma Association) Classification types A2, A3, and B1 fractures. Patients younger than 16 years were excluded because the displacement could be corrected with growth. We excluded type A1 fractures because they did not involve the distal radius. We excluded types B2 to C3 fractures because they were too complex for percutaneous fixation. Patients were also excluded if they had one of the following conditions: decline to participate; open fractures; ligament injuries; screw fixation alone; the use of an external fixator; associated carpal fractures, dislocations, or ulna fracture with dislocation; fractures 14 days old or more; multiple fractures; pathologic fractures; arthroscopic procedures; uncooperative adults, such as those with dementias; and infections, diabetes, rheumatoid arthritis, or gout. Preoperative X-rays and CT images were taken in all patients. All operations were performed by the same senior surgeon.

### Surgical technique

The operation was performed under brachial plexus anesthesia without tourniquet control. First, we tried to reduce the fracture using a traction maneuver. If there was residual displacement or angulation, we reduced the fracture by percutaneous K-wire leverage.

**Fig. 1:**
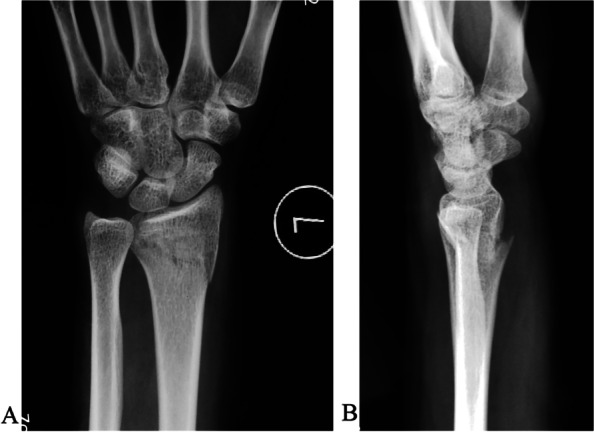
A type B1 distal radial fracture. **A** Posteroanterior view. **B** Lateral view

**Fig. 2:**
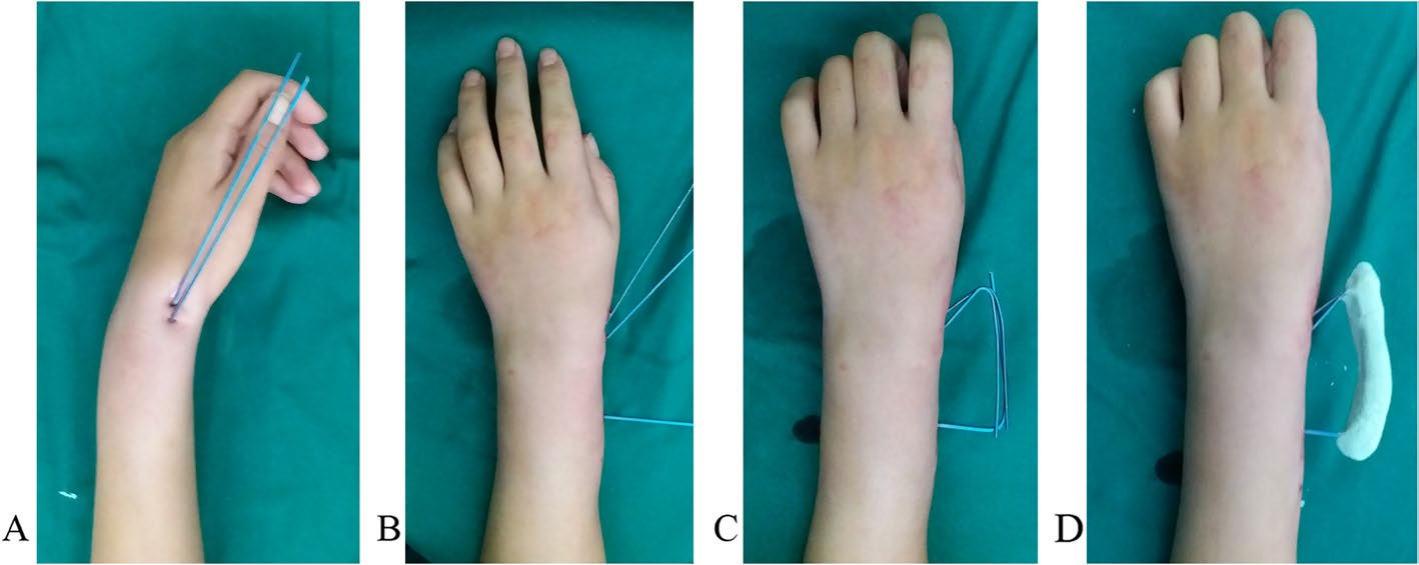
An 18-year-old male patient is treated with K-wires. **A** Two oblique K-wires are introduced through the radial styloid. **B** the third K-wire is introduced into the proximal fragment. **C** All K-wire ends are bent toward the fracture site. **D** Bone cement is mounted

**Fig. 3:**
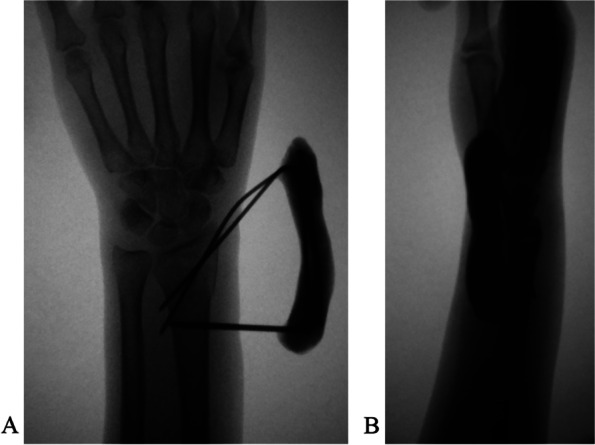
Reduction and wire position are checked under fluoroscopy. **A** Anteroposterior view. **B** lateral view

The radial styloid process was used as the anatomical landmark to determine K-wire insertion. For a type B1 fracture (Fig. 1A, B), we introduced one or more obliquely oriented K-wires (1.5 to 2.0 mm in diameter) from distally to proximally (Fig. 2A). Then, we introduced one or two transverse bicortical K-wires into the proximal fragment (Fig. 2B). Acceptable reduction and wire position were confirmed using fluoroscopy. The K-wires were bent toward the fracture site about 1.5 cm away from the skin (Fig. 2C). We mixed Monomer (liquid) and polymer (powder) of bone cement (Single dose 40 g US$170; PALACOS®, Hanau, Germany). When the bone cement viscosity changed over time from a runny liquid into a dough-like state, we applied it to the bent K-wires ends and waited for it to harden into a solid material (Fig. 2D). Thus, we created a cemented K-wire frame to prevent wire migration. Acceptable reduction and wire position were confirmed using fluoroscopy (Fig. 3A, B).

**Fig. 4:**
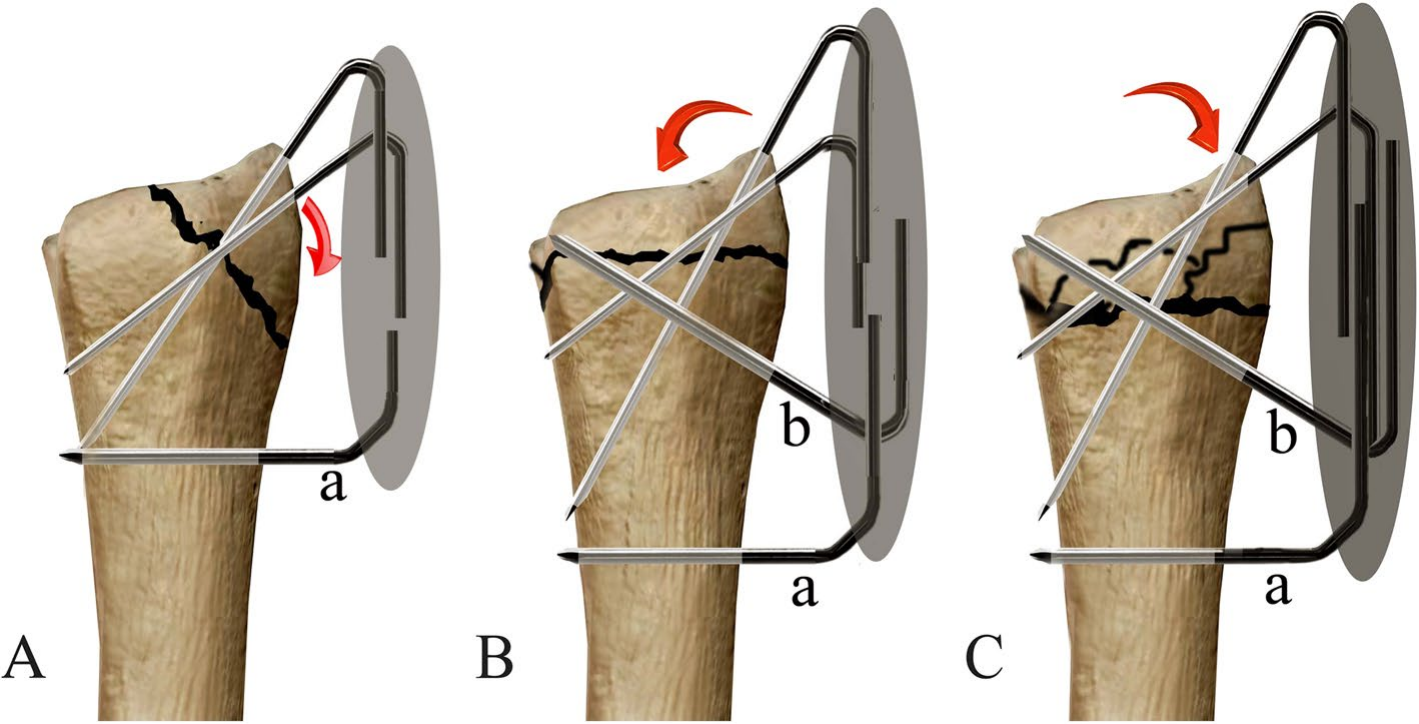
**A**. A type B1 fracture with a smaller distal fragment and oblique fracture line. The transverse K-wire (a) prevents radioproximal redisplacment of fragment and radioproximal wire shift. The arrow shows the direction that the fragment and K-wire tend to displace. **B**. A type A2 fracture with a volar displaced fragment is reduced and fixed. The proximal transverse K-wire (a) and oblique K-wire (b) prevent the fragment rotated volarly. The arrow shows the direction that the fragment and K-wire tend to displace. **C**. A type A3 fracture with a dorsally displaced fragment is reduced and fixed. The K-wires (a and b) prevent the fragment rotated dorsally. The arrow shows the direction that the fragment and K-wire tend to displace

**Fig. 5:**
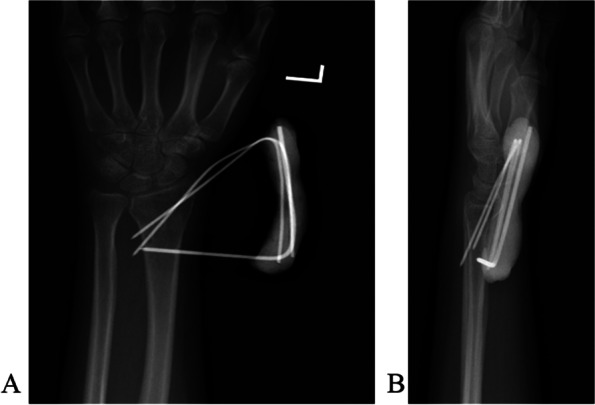
Bone healing after 4 weeks. **A** Anteroposterior view. **B** Lateral view

**Fig. 6:**
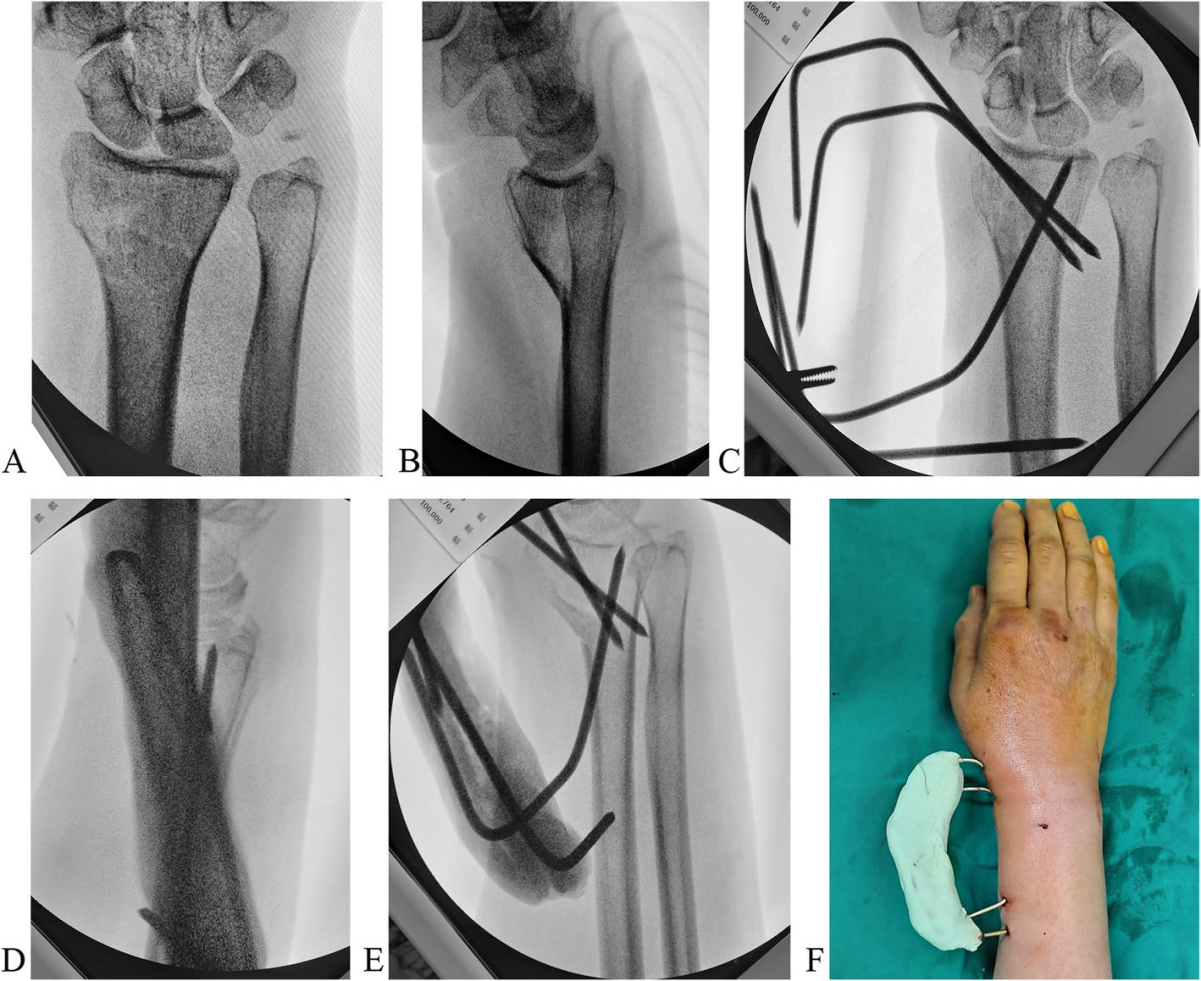
A type A2 fracture with a volar displaced fragment. **A** Preoperative anteroposterior view. **B** Lateral view. **C** Postoperative anteroposterior view. **D** Lateral view. **E** Oblique view. **F** All K-wire ends are mounted with bone cement to form a cemented frame

**Fig. 7:**
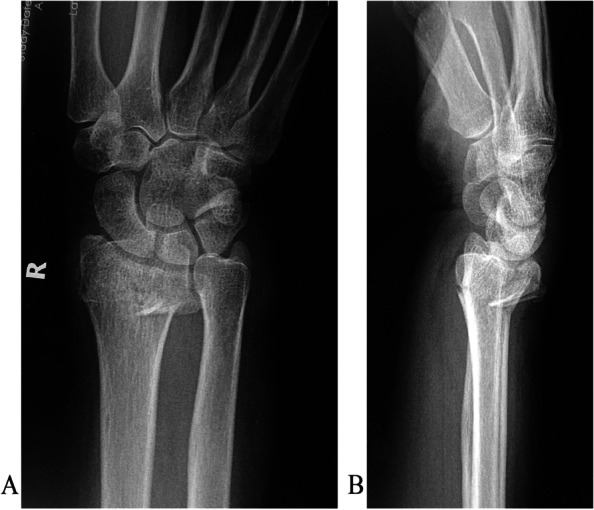
A type A3 distal radial fracture with dorsal fragments and without the dorsal support. **A** Posteroanterior view. **B** Lateral view

**Fig. 8:**
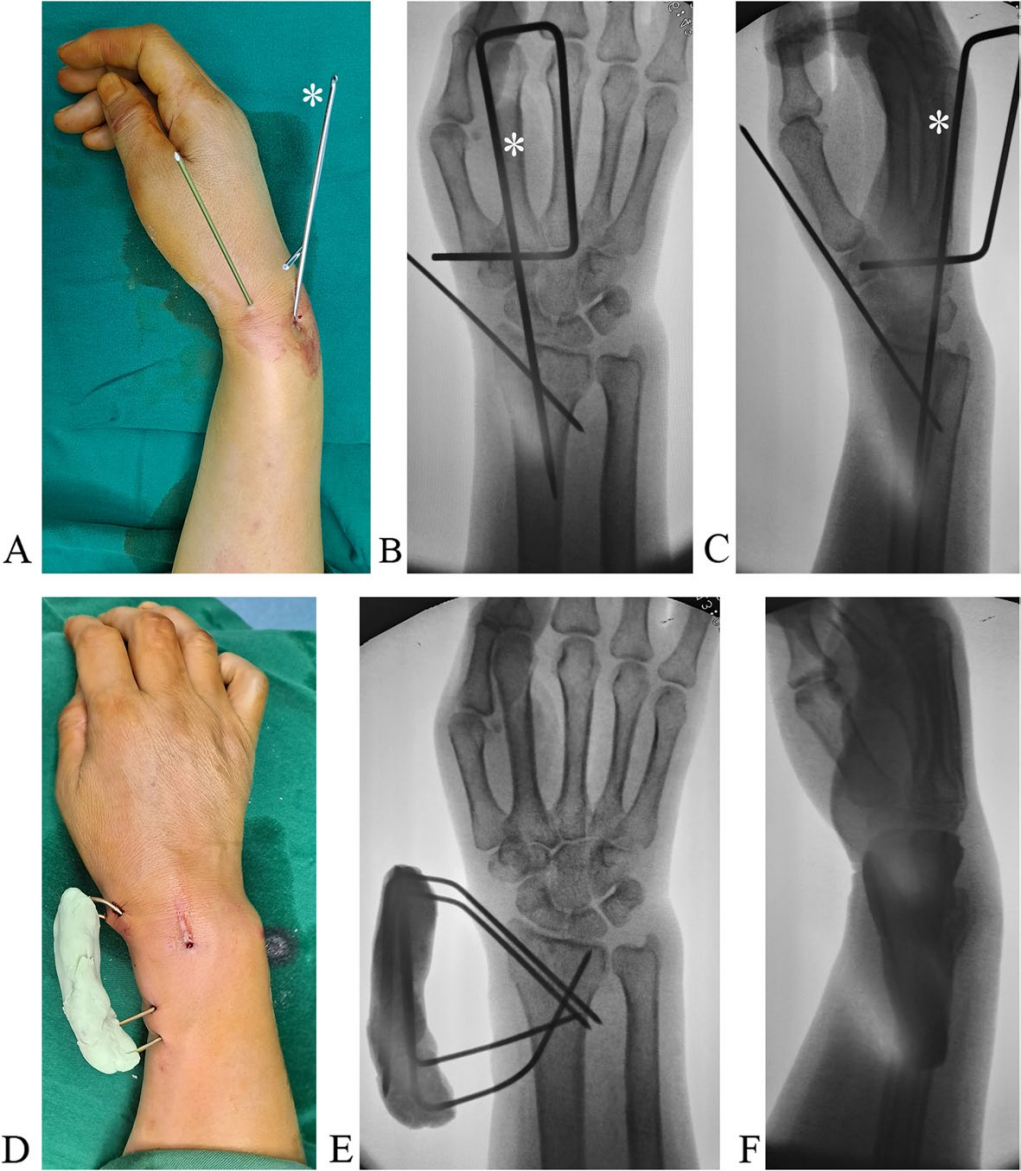
**A** Reduction is achieved with maneuver and Kapandji technique (* showing reduction K-wire), and the distal fragment is fixed with a K-wire. **B** Reduction on anteroposterior view. **C** Reduction on the lateral view. **D** More K-wires are added, and all K-wire ends are mounted with bone cement to form a cemented frame. E. Posteroanterior view. F. Lateral view

We used the same technique for a type B1 fracture with a small fragment (Fig. 4A). For types A1 and A2 fractures, we obliquely introduced one or two bicortical K-wires into the proximal fragment. Then, we introduced an oblique K-wire through the fracture site into the distal fragment and another transverse bicortical K-wires into the proximal fragment. The wire ends were bent and cemented as described above (Fig. 4B, C; Fig. 5A, B). Similar treatment was done in type A2 (Fig. 6A-F) and A3 fractures (Fig. 7A, B and Fig. 8A, B, C, D, E, F).

We performed a ballottement test with the wrist placed in the neutral, supination, and pronation. If there was any injury to the distal radioulnar joint, the ligament was repaired or reconstructed arthroscopically (*n* = 0).

### Postoperative management

After surgery, the K-wires and cement frame were protected using a commonly used dorsal short-arm splint or cast with the wrist in the neutral position. It allowed some degrees of wrist movement. After 4 weeks, the splint or cast was completely removed and wrist movement continued. When bone healing was achieved, the K-wires were cut off and removed.

### Outcome evaluation

Radiographic evaluation was performed on day 1 and every two weeks until bone healing had occurred. Palmar tilt was measured on the lateral view. Radial inclination, scapholunate gap, and ulnar variance were measured on posteroanterior radiograph [11]. Pin site infection was assessed based on the clinical symptoms [12]. Wrist pain intensity was assessed using the visual analog scale (VAS) [13].

At the final follow-up, active range of motion of the wrist was measured using a goniometer [14]. All measurements were compared to those on the opposite side. Grip strength of the hand was assessed using a Baseline hydraulic hand dynamometer (Fabrication Enterprises Inc., White Plains, NY) [15]. Isometric testing of pronation torque was assessed using McConkey method at 5 positions of rotation (90° of supination, 45° of supination, neutral, 45° of pronation, and 80° of pronation) [16]. In order to exclude any discrepancy between dominant and nondominant hand strength, we based the scores for analysis on the premise that the grip strength was 15% higher at dominant sides compared to the nondominant sides; and no correction was required for left-handed individuals [17]. Those measurements were compared to those on the opposite side. The patients rated their wrist pain and hand numbness using the 10-cm visual analogue scale [18]. We used the Mayo Wrist Score to assess wrist function (90–100, excellent; 80–90, good; 60–80, satisfactory; below 60, poor) [19]. Aesthetics and patient satisfaction were assessed using the 10-cm visual analog scale [20].

### Statistical analysis

Quantitative variables were described as mean and standard deviation for symmetric distribution or median and interquartile range for asymmetric distribution. We used Pearson’s chi-square test to compare categorical variables and Mann–Whitney U test to symmetric and asymmetric distribution. A *P* < 0.05 was considered statistical significance. The collected data were analyzed with the Statistical Package for Social Sciences 20.0 (SPSS, Inc., Chicago, Ill).

## Results

The mean age of the 78 patients was 37 years (range, 16 to 64 years). There were 47 male patients and 31 female patients. There were types A2 (*n* = 20), A3 (*n* = 36), and B1 (*n* = 22) DRFs (Table 1). We compared the X-rays taken immediately after surgery and bone healing. The results showed the fragments remained in place without significant redisplacement (Table 2) (*P* > 0.05). Pin site infection occurred in 3 patients, which healed by pin site care. Neither wire loosening nor fixation failure was found. Osteomyelitis was not observed in this series. Bone healing was achieved in all patients after a mean of 4.5 weeks (range, 4 to 8 weeks).

**Table 1:** Demographic data on 78 patients

Age (mean, range, yr)	37 (16—64)
Sex (m:f)	47:31
Dominant hand (n)	39
Cause (n)	
Fall	49
Road traffic accident	13
Sports	11
Others	5
AO/OTA (n)	
A2	10
A3	46
B1	22
Ulnar styloid fracture (n)	32
TBIO (mean, range, day)	4 (0—13)
Operative time (mean, range, minutes)	23 (18—41)
Number of K-wire (mean, range, n)	
Oblique	2.6 (2—4)
Transverse	1.3 (1—2)
Pin site infection (n)	3
Time of bone healing (mean, range, week)	4.5 (4—8)
Wrist pain (VAS; day 10; mean, range)	1 (0—2)

AO/OTA AO Foundation and Orthopaedic Trauma Association, *TBIO* Time between injury to operation

**Table 2:** Reduction maintenance measured immediately after surgery vs at bone healing

	Immediately after surgery	Bone healing	*P* value
Radial height (mm)	12.51 ± 3.22	12.45 ± 3.24	0.441
Palmar tilt (°)	11.19 ± 0.95	11.14 ± 0.94	0.069
Radial inclination (°)	18.7 ± 3.12	18.68 ± 3.12	0.159
Scapholunate gap (mm)	1.46 ± 0.16	1.45 ± 0.157	0.058
Ulnar variance (mm)	0.35 ± 0.53	0.364 ± 0.54	0.06
Articular stepoff > 2 mm (n)	0	1	-

Data are shown as mean ± standard deviation

The mean follow-up period was 27 months (range, 24 to 33 months) (Table 3). Active range of motion of the wrist and grip strength of the hand were similar to those on the opposite side. The mean score of wrist function was 97 (range, 91 to 100), including 66 excellent and 12 good results. The mean patient satisfaction score was 10 (range, 8 to 10).

**Table 3:** Outcomes at the final follow-up

	Mean	Range
Follow-up time (month)	27	24—33
Active ROM (°)		
Flexion	74	61—89
Extension	67	52—72
Radial deviation	30	25—38
Ulnar deviation	17	7—26
Pronation	80	67—95
Supination	85	74—97
Grip strength (%)^a^	98	94 -103
Supination torque (%)^b^		
90° of supination	92	83—98
45° of supination	95	92—98
Neutral	94	81—98
45° of pronation	94	87—97
80° of pronation	96	90—102
Wrist pain (MWS)	0	0—1
Numbness (VAS)^c^	0	0—1
Wrist function (VAS)	97	91—100
Aesthetics (VAS)	10	8 -10
Satisfaction (VAS)	10	8—10

*SD* Standard deviation, *ROM* Range of motion, *VAS* Visual analogue scale

^a^15% higher at dominant sides compared to the nondominant sides discrepancy, in which percentages show involved limb compared with opposite normal side

^b^Supination torque based on McConkey method

^c^distribution of all sensory nerves of the hand, *DASH* Disabilities of the arm, shoulder and hand scores, *MWS* Mayo Wrist Score

## Discussion

We find that the percutaneous fixation using a cemented K-wire frame may prevent radioproximal redisplacement and wire migration in type B1 DRFs; and in the sagittal plane, prevent rotational redisplacement in types A2 and A3 DRFs. Moreover, the system effectively maintains the radius height. The minimally invasive technique is reliable with minimal complications and produces satisfactory outcomes.

DRFs are often caused by a fall on an outstretched hand [21]. The treatment strategies for DRFs are generally controversial [22]. Surgeon preferences, fracture types, comorbidities, complications, and patient factors (lifestyle, age, mental attitude, social support, comorbid conditions, and compliance and adherence) influence the options [23]. Usually, types A2, A3, B1, and B2 (AO/OTA classification) fractures can be treated with a splint, cast, plate and screw system, external fixator, or K-wires, or a combination of them [3, 24, 25]. A splint or plaster cast is usually used for up to 4 to 6 weeks to immobilize the fracture in place while healing occurs [26]. Open reduction and internal fixation may be required for unstable fractures, but the incidences of complication (fixation failure, tendon irritation, and tendon rupture, and wound infection) are reported as high as 3% to 27%, depending on the fixation technique used [27, 28].

Franceschi et al. [29] conducted a systematic review of plating versus pinning for the treatment of DRFs. They found plating is associated with higher incidences of plate-induced tendon irritation (wrist pain, carpal tunnel syndrome, tendonitis, and tenosynovitis). In comparison, pinning is associated with higher incidences of tendon and neurovascular injuries (temporary paraesthesia, nerve irritation, superficial infections), wire migration, and fixation failure due to improper wire placement. However, the comparison based on types A1 to C3 DRFs may not reflect actual outcomes because plates are more likely to be used for complex fractures (types B3 to C3). Nevertheless, reducing the pinning-induced complications is a laudable goal.

There are many percutaneous pinning techniques (e.g., the use of two K-wires, the use of three or more K-wires, and Kapandji-technique) for DRFs [4]. Typically, one or two K-wires are introduced into the radial styloid process through the radial styloid window. More K-wires can be added between the1 and 2 compartments, 2 and 3 compartments, and 4 and 5 compartments. However, identifying the proper insertion sites and achieving optimal wire placement are difficult [6]. Moreover, oblique and cross pinning can effectively stabilize transverse fractures because stability depends on the intact of the proximal fragment [30]. However, the support is compromised in oblique and comminuted fractures [7]. According to the earlier anatomical studies, there are no important structures in the radial aspect of distal 1/3 radius, where K-wires can be safely introduced [31]. A cement block connects all K-wire ends together to form a frame, which prevents wire migration. Therefore, a relatively rigid fixation can be achieved, which allows for early joint movement in a splint.

In type B1 DRFs, the distal fragment tends to redisplaced radioproximally, and the distal K-wires tend to shift radioproximally due to a single cortical wall being engaged (Fig. 4A). In types A2 and A3 DRFs, the distal fragments tend to redisplaced due to oblique fracture lines or comminuted patterns. The two proximal K-wires not only prevent redisplacement of the fragment and wire shift, but also reinforce the fixation (Fig. 4B, C). Similar to an external fixator, the frame maintains the radial height. Bain et al. [32] studies the common DRF patterns (dorsal, volar, and radial styloid patterns) and found the ligamentous attachments to the volar carpus were well preserved. Thus, the fracture fragments can be conceptualized as an osseo-ligamentous unit, providing additional stability even though the K-wires do not hold all fragments.

The advantages of our technique are a minimally invasive procedure that achieves acceptable stability. Compared to the nonsurgical treatments, out fixation is stable and prevents redisplacement. Compared to the conventional pinning, out technique prevents wire migration by securing all wire ends together. Compared to plating, our technique is a minimally invasive procedure that avoids wound complications and allows early implant removal. Compared to conventional external fixators, our system is easy to install and much cheaper.

We used the radial styloid process as the anatomical landmark to prevent injuries to the superficial branch of the radial nerve, tendons, and vessels. Wire placement is relatively safe. The procedures are easy to perform, and the fixation is relatively rigid. It prevents bone shortening and allows early wrist movement. The disadvantage is the risk of iatrogenic injuries to the tendons, nerves, and vessels, although in our experience the incidence is much lower than that of the conventional pinning technique. In a systematic review, Karantana et al. [33] found the incidence of pin site infection was 8% (range, 0% to 15%), which can be treated by pin site care and rarely requires antibiotics or early wire removal. Solari et al. [34] retrospectively reviewed 200 patients treated with 369 percutaneous K-wires. Pin site infection was diagnosed in 9 patients (5%), and the wire survival rate was 99.5%. Pin loosening is associated with a high risk of pin site infection [35], which can be decreased by securing all wire ends to a cemented frame.

The main indication for our technique is DRFs involving the radiocarpal articular surface or not, including oblique, die-punch, and comminution DRFs, especially AO/OTA classification types A2, A3, B1, and B2 DRFs. Contraindications are (1) severely comminuted DRFs, especially at the pin site areas, since wire migration may lead to redisplacement; (2) DRFs with combined tendon, nerve, artery, or ligament injuries requiring extensive exposure; and (3) old fractures requiring open reduction and grafting. We do not consider the technique to be a standard treatment because more rigid fixation can be achieved with a locking plate. Therefore, our technique is an adjunct to the conventional treatment for DRFs, especially for special cases or countries with limited resources. Moreover, our technique in combination with other techniques may be a good choice for complex DRFs.

The major limitation of the study is the lack of kinematics that require further study. Surgeon preference, experience, and abilities may influence wire configuration and placement. The operations and assessments were done at different times, and surgeons’ experience improved over time, which may influence determination of the technical effect.

## Conclusion

Percutaneous fixation with cemented K-wire frame is a safe and preferred alternative for the treatment of types A2, A3, and B1 DRFs. The frame provides support to prevent redisplacement and wire migration. The technique is a minimally invasive procedure, which is easy to perform.

## Supplementary Information

**Additional file** **1.** Patients’ preoperative and postoperative data.

## Data Availability

Data is available on a supplementary file entitled “rare data Distal Radius Fractures” and also datasets used and analyzed during the current study are available from the corresponding author upon reasonable request.
